# *Enterocytozoon bieneusi* in Wild Rats and Shrews from Zhejiang Province, China: Occurrence, Genetic Characterization, and Potential for Zoonotic Transmission

**DOI:** 10.3390/microorganisms12040811

**Published:** 2024-04-17

**Authors:** Ting Zhang, Kuai Yu, Junchen Xu, Wenjie Cao, Yiqing Wang, Jiayan Wang, Liyuting Zhou, Jiani Chen, Huicong Huang, Wei Zhao

**Affiliations:** School of Basic Medical Sciences, Wenzhou Medical University, Wenzhou 325035, China; 15267752993@163.com (T.Z.); yk428316@163.com (K.Y.); 18767080902@163.com (J.X.); jefery628@hotmail.com (W.C.); wyq0238@163.com (Y.W.); w15267752665@126.com (J.W.); hippo107@126.com (L.Z.); chenaltair@163.com (J.C.)

**Keywords:** zoonotic, *Enterocytozoon bieneusi*, rodents, shrews, China

## Abstract

Globally, *Enterocytozoon bieneusi* has been detected in humans and various animal hosts. Wild rats and shrews have the potential to act as carriers of *E. bieneusi*, facilitating the parasite’s transmission to humans and domestic animals. We aimed to investigate the prevalence of *E. bieneusi* in 652 wild rats and shrews from Zhejiang Province, China, by amplifying the internal transcribed spacer (ITS) region of rDNA through polymerase chain reaction (PCR). To determine animal species, we amplified the *Cytochrome b* (*Cyt-b)* gene in their fecal DNA using PCR. Furthermore, we determined the genotype of *E. bieneusi* by amplifying the ITS region of rDNA through PCR. Genetic traits and zoonotic potential were evaluated using similarity and phylogenetic analyses. *Suncus murinus* (n = 282) and five rat species, *Rattus losea* (n = 18), *Apodemus agrarius* (n = 36), *Rattus tanezumi* (n = 86), *Rattus norvegicus* (n = 155), and *Niviventer niviventer* (n = 75), were identified. The average infection rate of *E. bieneusi* was 14.1% (92/652) with 18.1% (51/282) in *S. murinus* and 11.1% (41/370) in rats (27.8% in *R. losea*, 22.2% in *A. agrarius,* 10.5% in *R. tanezumi*, 8.4% in *R. norvegicus*, and 8.0% in *N. niviventer*). Thirty-three genotypes were identified, including 16 known genotypes. The most commonly known genotypes were HNR-VI (n = 47) and Peru11 (n = 6). Type IV, KIN-1, SHW7, and HNPL-II were each found in two samples, while Macaque4, CH5, K, Henan-III, Henan-V, HNP-II, HNPL-I, HNPL-III, HNHZ-II, and HNHZ-III were each found in one sample. Additionally, 17 novel genotypes were discovered: WZR-VIII (n = 5), WZR-I to WZR-VII, WZR-IX to WZR-XII, and WZSH-I to WZSH-V (n = 1 each). Those 33 genotypes were divided into three groups: Group 1 (n = 25), Group 2 (n = 3), and Group 13 (n = 5). The initial report underscores the extensive occurrence and notable genetic diversity of *E. bieneusi* in wild rats and shrews from Zhejiang province, China. These results suggest that these animals play a pivotal role in the transmission of *E. bieneusi*. Furthermore, animals carrying the zoonotic genotypes of *E. bieneusi* pose a serious threat to residents.

## 1. Introduction

Microsporidia are a group of obligate intracellular pathogens consisting of approximately 1600 species distributed across 218 genera [1]. Previously categorized as parasites, they are now recognized as close relatives of fungi with a broad host range, encompassing all major animal groups, including humans [2]. Since microsporidia were discovered to be capable of infecting humans, 10 genera and 17 species of microsporidia have been reported to cause human infections [3]. Among them, *Enterocytozoon bieneusi* is the most common and gained public attention in 1985 when it was identified as a comorbid condition in AIDS patients [4]. Subsequently, it has been frequently reported in AIDS patients and is now recognized as one of the most common pathogens causing intestinal diarrhea in this population [5]. More recently, it has been detected in individuals with normal immune function where chronic diarrhea is the primary clinical symptom, although infections can be asymptomatic [6]. Humans typically become infected with *E. bieneusi* by swallowing the parasite’s infectious spores, which are excreted in feces and easily spread between hosts via the fecal–oral route [7]. These spores can be found in various sources, including water, soil, and environmental surfaces with fecal contamination and fruits and vegetables irrigated with improperly treated water [7,8]. *E. bieneusi* has garnered significant attention due to its ability to be transmitted through water and its wide host range. The National Institute of Allergy and Infectious Diseases has classified it as a Class B priority pathogen, while the US Environmental Protection Agency has identified it as a contaminant candidate for aquatic microorganisms [9,10]. To effectively contain the spread of *E. bieneusi* in humans, it is imperative to identify its infectious agents and modes of transmission. The cornerstone of these efforts is the accurate identification of *E. bieneusi*.

Molecular diagnostic technology can trace the source of infections and deduce the transmission route and dynamics of *E. bieneusi*. Molecular typing tools are used to identify different genotypes of *E. bieneusi* based on polymorphisms in the ribosomal internal transcribed spacer (ITS) nucleotide sequence [11]. This tool has enhanced our understanding of the distribution and zoonotic potential of *E. bieneusi* genotypes in humans and various animals. Over 850 distinct genotypes of *E. bieneusi* have been identified in humans and animals, and this number is still increasing [12]. At least 126 of these genotypes have been recorded exclusively in humans, while 58 of them were zoonotic (present in both humans and animals) [12]. We can evaluate the zoonotic transmission capacity of genotypes using evolutionary analysis. These genotypes are divided into 13 groups [13]. Among them, the first and second groups have a greater possibility of zoonotic transmission, while the other groups show a level of host specificity [12]. In animals, the genotypes identified in pigs are almost all in the first group, while the genotypes in cows and sheep are mostly in the second group [14,15]. Additionally, some host genotypes may have unique groups, providing important references for understanding the threats of the genotypes of different host sources to humans [16]. However, the precise role of each host in the transmission of the disease remains unclear. Therefore, it is crucial to monitor and investigate a range of hosts, particularly those in contact with humans, to effectively manage the outbreak prevalence of *E. bieneusi*.

Rodentia is the most populous and diverse order of mammals globally, encompassing 1780 species. The worldwide combined population of brown rats (*Rattus norvegicus*) and house mice (*Mus domesticus*)—two examples of wild rodents—totals over 7 billion, which is almost equal to (or exceeds) the human population [17]. Rodents can serve as carriers of *E. bieneusi*, enabling its spread to humans and other animals in both rural and urban settings. Notably, the estimated *E. bieneusi* infection rate in rodents is 17%, which is significantly higher than the rate of 6.6% observed in humans [6,17]. Furthermore, typing data reveals that these rodents carry over 100 genotypes of *E. bieneusi*, significantly overlapping with the genotypes found in humans [18]. These statistics suggest that rodents play a pivotal role in the transmission of *E. bieneusi* to humans and should not be overlooked in efforts to combat this parasite.

In China, *E. bieneusi* infections have been the subject of extensive research, with 148 infected hosts and 22 infected rodent species identified [7,18,19]. These studies have spanned across five provinces, revealing infection rates ranging from 3.6% to 35.1% [18,19,20]. However, only one study has specifically looked at the infection of *E. bieneusi* in wild rodents in Zhejiang Province, where *E. bieneusi* was identified in humans and in farmed and wild animals [21]. The current study aimed to assess the zoonotic potential of *E. bieneusi* isolates at the genotype level by exploring their prevalence in rodents across several regions of Zhejiang Province.

## 2. Methods

### 2.1. Ethical Approval

The current study was carried out in accordance with the Chinese Laboratory Animal Administration Act (1988), which dictates the ethical handling and use of animals in scientific research. The research protocols were meticulously reviewed and approved by the Research Ethics Committee of Wenzhou Medical University (SCILLSC-2021-01, 10 March 2020).

### 2.2. Sample Collection

From 1 April to 31 October of 2023, a total of 652 wild rodents and shrews were captured across three distinct locations within rural areas immediately adjacent to human habitations in Zhejiang Province, China. These included 94 animals caught in Yueqing, 170 in Yongjia, and 388 in Rui’an. The animals were lured into cage traps using deep-fried dough sticks. The traps were placed at various designated locations, with approximately 50 set out each evening and retrieved before sunrise. The traps were placed every 5 m along a linear transect. Within 48 h of capture, all rodents were transported to a laboratory and euthanized via CO_2_ inhalation. We noted the time and region of each collection. Immediately after that, we obtained a fresh fecal sample (500 mg) from the rectal and intestinal material of each rodent. The samples were then stored in ice boxes and transported to the laboratory, where DNA was extracted within a week.

### 2.3. DNA Extraction

Following the manufacturer’s guidelines, genomic DNA was isolated from each processed sample (200 mg) using the QIAamp DNA Mini Stool Kit (Qiagen, Hilden, Germany). To achieve a significant quantity of DNA, the lysate temperature was raised to 95 °C. Before polymerase chain reaction (PCR) analysis, DNA was reconstituted in 200 µL of AE elution buffer (provided in the kit) and stored at −20 °C.

### 2.4. Identification of Rodent and Shrew Species

Rodent and shrew species were identified by amplifying a defined gene (*cytb* with 421 bp) extracted from fecal DNA using PCR. The primer design and PCR conditions followed the guidelines provided by Verma and Singh (2003) [22]. Each PCR reaction involved 35 cycles, including denaturation at 94 °C for 30 s, annealing at 51 °C for 30 s, and extension at 72 °C for 30 s. Before the cycling steps, an initial denaturation was performed at 94 °C for 5 min, followed by a final extension at 72 °C for 5 min.

### 2.5. Genotyping of E. bieneusi

Nested PCR was used to amplify the ITS region and identify the genotype of *E. bieneusi*. TaKaRa Taq DNA Polymerase was employed, along with the genotype BEB6 DNA from deer as a positive control and 2 μL distilled water as a negative control. Buckholt et al. designed the primers and cycle parameters [23]. The PCR products were analyzed using 1.5% agarose gel electrophoresis, followed by visualization with DNAGREEN staining (Tiandz Inc., Beijing, China).

### 2.6. DNA Sequencing and Analysis

The PCR products that tested positive for *E. bieneusi* underwent bidirectional sequencing (performed by Sangon Biotech Co., Ltd., Shanghai, China). Additional PCR products were sequenced as necessary. For the genotyping of the *E. bieneusi* isolates, we utilized the Basic Local Alignment Search Tool (BLAST) and ClustalX 1.83 software. This involved comparing the identified nucleotide sequences with published GenBank sequences. The genotypes were labeled according to the established nomenclature based on the 243 bp of the ITS region of *E. bieneusi*.

### 2.7. Phylogenetic Analysis

A phylogenetic tree was constructed using the Mega 7 software, employing the maximum likelihood method and Tamura–Nei parameter model. Additionally, 1000 replicates were conducted to assess the relationship between the genotypes identified in this study and to confirm the gene group.

### 2.8. Statistical Analyses

All the data presented were analyzed using the SPSS software (Version 22.0, SPSS Inc., Chicago, IL, USA). To assess the differences in *E. bieneusi* prevalence among rodent species, regions, genders, and seasons, the chi-square test was used for each of these variables. Statistical significance was considered at a threshold level of *p* ≤ 0.05.

### 2.9. Nucleotide Sequence Accession Numbers

The nucleotide sequences of *E. bieneusi* genotypes identified in the present study have been submitted to the GenBank database with the accession numbers PP391796 to PP391828.

## 3. Results

### 3.1. Study Population

In this study, we used PCR and sequencing analysis of the *cytb* gene to identify the species of the animals investigated. Out of 652 samples, 370 were identified as five wild rat species, including *R. norvegicus* (n = 155), *R. tanezumi* (n = 86), *Niviventer niviventer* (n = 75), *Apodemus agrarius* (n = 36), and *R. losea* (n = 18). The remaining 282 samples were identified as *Suncus murinus*. Most of the samples (42.0%, 274/652) were collected during the summer, 30.1% (196/652) in autumn, 27.3% (182/652) in spring, and none in the winter. The rodents’ sex distribution was 45.2% (295/652) female and 54.8% (357/652) male (Table 1).

### 3.2. Prevalence of E. bieneusi

We detected *E. bieneusi* in 14.1% (92/652) of the samples with 11.1% (41/370) in wild rats and 18.1% (51/282) in *S. murinus.* Among the wild rats, *R. losea* had the highest prevalence rate of *E. bieneusi* (5/18, 27.8%), followed by *A. agrarius* (8/36, 22.2%), *R. tanezumi* (9/86, 10.5%), *R. norvegicus* (13/155, 8.4%), and *N. niviventer* (6/75, 8.0%) (Table 1). There are significant differences in infection rates among those animal species (χ^2^ = 15.848, df = 5, *p* = 0.007). Out of the three areas, the animals from Yueqing had the highest infection rate of 25.5% (24/94), followed by those from Yongjia with 20.6% (35/170), and those from Rui’an had the lowest infection rate of 8.5% (33/388) (Table 1).

The infection rates also varied significantly depending on the geographic location (χ^2^ = 26.063, df = 2, *p* < 0.001). Meanwhile, the prevalence of *E. bieneusi* in animals collected in summer (22.6%; 62/274) was significantly higher than those collected in spring (12.1%; 22/182) and autumn (4.1%; 8/196) (χ^2^ = 33.28; df = 2; *p* < 0.001). The incidence of *E. bieneusi* was significantly higher in females (23.1%; 68/295) than in males (6.7%; 24/357) (χ^2^ = 35.533; df = 1; *p* < 0.001) (Table 1).

### 3.3. Characterization and Distribution of the Genotypes of E. bieneusi

ITS sequencing of 92 *E. bieneusi* isolates identified 33 genotypes, including 16 known genotypes (HNR-VI, Peru11, Type IV, KIN-1, SHW7, HNPL-II, Macaque4, CH5, K, Henan-III, Henan-V, HNP-II, HNPL-I, HNPL-III, HNHZ-II, and HNHZ-III) and 17 novel genotypes (WZR-I to WZR-XII and WZSH-I to WZSH-V). Amongst them, HNR-VI (44.2%, 47/92) dominated, followed by Peru11 (6.5%, 6/92) and WZR-VIII (5.4%, 5/92), while the other genotypes were present at low frequencies with Type IV, KIN-1, SHW7, and HNPL-II found in two samples and the remaining genotypes, Macaque4, CH5, K, Henan-III, Henan-V, HNPL-I, HNP-II, HNPL-III, HNHZ-II, HNHZ-III, WZR-I to XII, and WZSH-I to V, present in one sample (Table 2).

Except for WZR-XII, which had the largest similarity (81.4%) with the genotype NESH2 (KP732476) isolated from sheep from China, the novel genotypes identified here presented one to six or nine base differences compared with previously reported genotypes (Table 3).

The distribution of *E. bieneusi* genotypes among *S. murinus* and different rat species is diverse (Table 2). HNR-VI was found in both *S. murinus* and wild rats, and Peru11, Type IV, KIN-1, Macaque4, Henan-V, HNHZ-III, HNPL-I, HNPL-II, HNPL-III, CH5, and WZSH-I to V were only found in *S. murinus*, while SHW7, K, Henan-III, HNP-II, HNHZ-II, and WZR-I to WZR-XII were only found in wild rats. HNHZ-II, SHW7, and K were only found in *R. norvegicus*; Henan-III only in *R. tanezumi*; HNP-II and WZR-IX to WZR-XII only in *A. agrarius*; and WZR-VIII in both *N. niviventer* and *A. agrarius* (Table 2). The genotypes were divided into groups based on sex, season, and geographical region (Table 2).

### 3.4. Phylogenetic Analysis

**Figure 1:**
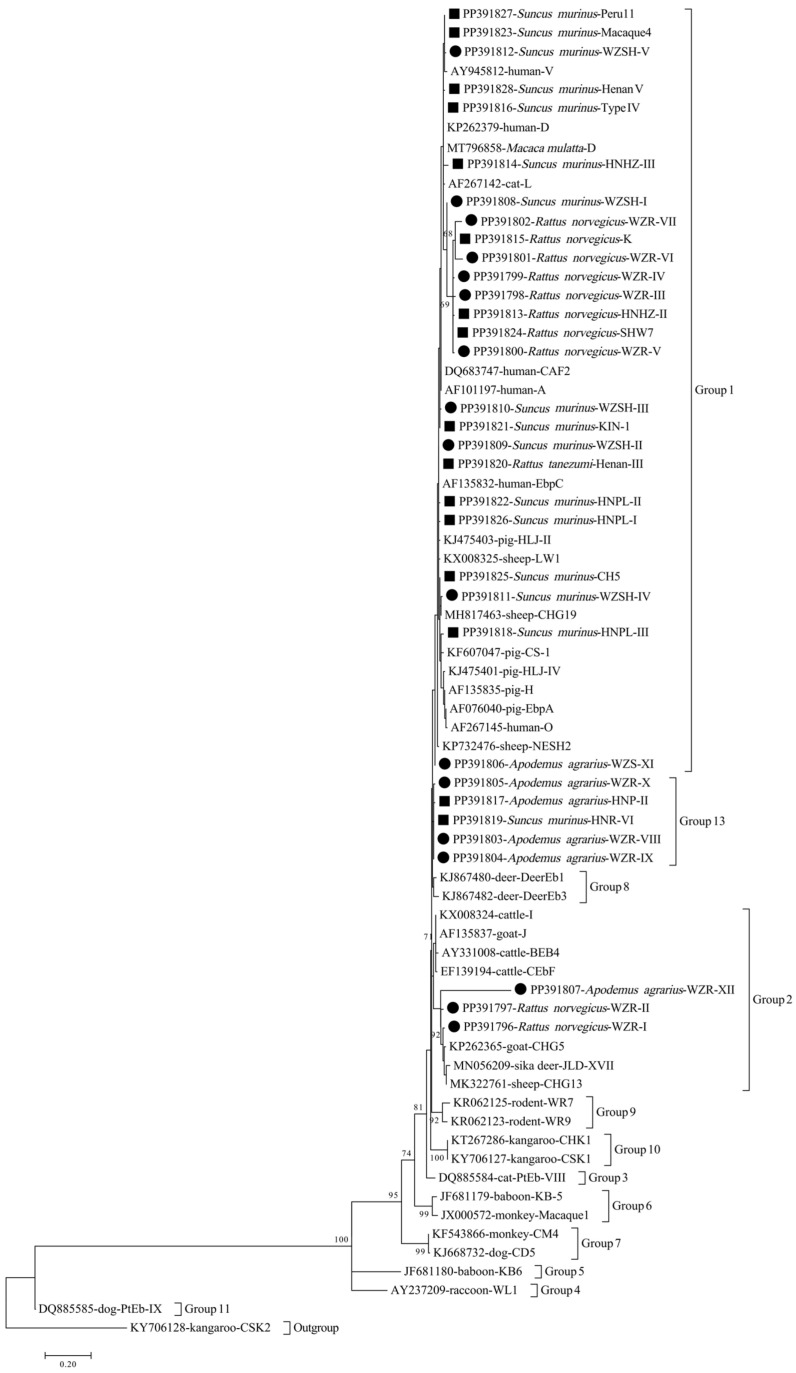
Phylogenetic tree of *E. bieneusi* genotypes based on ITS sequences. The genetic relationships among the various *E. bieneusi* genotypes were established through a phylogenetic tree generated using the maximum likelihood method. The Tamura–Nei parameter model was utilized to calculate evolutionary distances. The bootstrap values annotated above the nodes are based on the results of 1000 repetitions, and only values greater than 50 are shown. In this tree, genotypes are denoted by black squares and circles to distinguish between known and novel sequences identified in this study.

Phylogenetic analysis of the ITS region of *E. bieneusi* divided the identified genotypes into three distinct groups: Group 1 (n = 25), Group 2 (n = 3), and Group 13 (n = 5) (Figure 1).

## 4. Discussion

This study reports the identification of *E. bieneusi* in rodents and shrews in Zhejiang Province, located in the southeastern region of China. To date, 23 studies from eight different countries have found *E. bieneusi* in rodents, with a prevalence of 1.1–100.0% [12,19]. Notably, there were geographical variations in the average prevalence of *E. bieneusi* in rodents: 14.9% (10/67) in Peru [24], 38.9% (121/311) in Poland [25], 18.0% (85/472) in the United States [26,27,28], 10.7% (31/289) at the Czech Republic and Germany border [29], 13.9% (1021/7336) in China [13,18,19,20], 13.0% (55/423) in Japan [30], and 1.1% (3/280) in Slovakia [31]. These studies also reported variation in the prevalence of *E. bieneusi* infections across species: 27.3% in *Apodemus* spp., 3.6% in Chinchillas, 87.5% in guinea pigs, 48.3% in prairie dogs, 39.1% in vole, 24.3% in hamsters, 16.7–42.9% in squirrels, 4.0–36.4% in wild rats, 3.6–71.4% in chipmunks, and 1.1–87.5% in mice [13,18,19,20]. Those animals can be categorized as wildlife, pets, zoo animals, experimental animals, and agricultural animals. Compared with pets and experimental animals, wild rodents have a higher infection rate of *E. bieneusi* [18]. Notably, except for China and the United States, only one study was performed in each of the other countries, and thus further large-scale surveillance studies should be conducted to ascertain these findings.

Of the 33 identified *E. bieneusi* genotypes, HNR-VI was the most prevalent, occurring in more than half of the isolates (51.1%; 47/92). This genotype is widely distributed and has been found in all sampled animal populations except for *A. agrarius*. Initially discovered in Asiatic brush-tailed porcupines, HNR-VI has since been identified as the dominant genotype in civets in the same area [12,18,32]. Furthermore, there are some synonymous names, e.g., NMGH1 identified in horses, MJ14 (MK348513) in *Arctictis binturong* and pigs, PL2 in civets, and CPB19 (OQ534110) in giant pandas [14,33,34]. Although no human infections have been reported with this genotype, the above data suggest that the HNR-VI genotype has a broad host range. It is anticipated that, with further research, the true host range of this genotype will be discovered.

Among the 15 identified genotypes, five (Peru11, Type IV, KIN-1, Henan-III, Henan-V, and K) are known to be zoonotic. Peru11 and Type IV are frequently detected in humans and have a broad host range, including non-human primates, domestic animals, and birds [12]. These two genotypes were found in 8.7% (8/92) of the animals surveyed, indicating a high potential for transmission from infected animals to both humans and other animals. In contrast, KIN-1, Henan-III, Henan-V, and K are less common in humans. For example, KIN-1 has been identified in a healthy Cameroonian, Henan-III and Henan-V in a person infected with HIV, and K in one person from China (OR827708) [35,36]. However, they have also been detected in a variety of hosts. KIN-1 has been identified in pigs, cattle, sheep, goat, deer, civet, Asiatic brush-tailed porcupines, bamboo rats, and captive wild animals [18,32,37,38,39,40,41]. Henan-III has been identified in pigs, whooper swans, pet snakes, Asiatic brush-tailed porcupines, bamboo rats, civets, and wild rhesus macaques [18,32,42,43,44,45]. Henan-V has been found in captive snakes, dogs, and macaques [41,46,47]. K has been found in wild rats (*Leopoldamys edwardsi* and *Berylmys bowersi*) [48]. Additionally, some of these genotypes were found on environmental surfaces and in vegetables and fruits [49]. The detection of these genotypes in horticultural products monitored in this study expands their host range and highlights their potential public health significance. These findings suggest that small rodents may play a significant role in maintaining the cycling of *E. bieneusi* among humans, livestock, wildlife, and the environment.

The remaining nine known genotypes have not been found in humans, and their zoonotic potential remains uncertain. However, these genotypes have been identified in multiple animal hosts. For example, SHW7 and HNPL-III have been identified in civets and bamboo rats, HNPL-I and HNPL-II in civets, Macaque4 in macaques and civets, CH5 and HNP-II in pigs and cattle, and HNHZ-II and HNHZ-III in Asiatic brush-tailed porcupines [14,18,32,37,38,42]. All nine genotypes mentioned above have been identified in *S. murinus* here, suggesting that these genotypes have a wide range of animal hosts and may cause cross-species transmission. This also suggests that *S. murinus* may hide more genotypes. Therefore, further investigation should be carried out to determine the true host range of these genotypes and to analyze the genotype distribution of *S. murinus*.

In this study, 17 novel genotypes were identified, of which 12 belonged to Group 1 or Group 2, which are the two most prevalent and complicated groups, with over 600 genotypes [16]. The genotypes in these groups have been identified in several hosts, including humans, and possess a high potential for cross-species and zoonotic transmission. This suggests that the 12 novel genotypes may have a wider host range and could be capable of infecting humans. However, more research is required to confirm this.

Genotypes WZR-VIII to WZR-XI were grouped in Group 13 with HNR-VI, which was the most dominant genotype identified in the present study. It is inferred that these three genotypes may share genetic similarities with HNR-VI and may have evolved from it. Furthermore, the genotypes in Group 13 included other genotypes, such as HNP-II in pigs and SCR06 in rabbits, suggesting that the genotypes within Group 13 are not exclusive to rodents [13,14]. However, it becomes increasingly apparent that solely relying on ITS sequence data is inadequate for providing a robust phylogenetic signal across the entire tree [12]. Consequently, future studies necessitate the utilization of additional genetic markers to comprehensively comprehend the genetic affinities among these genotypes and to assess their likelihood of cross-species transmission and zoonosis.

## 5. Conclusions

Our research revealed a concerning rate of *E. bieneusi* infections among various wild rat and shrew species in Zhejiang, China. The identification of zoonotic genotypes of *E. bieneusi*, including Peru11, Type IV, KIN-1, Henan-III, and K, in these animals, highlights the potential public health risks in this region. Therefore, it is crucial to raise awareness about the risks posed by these animals and take measures to curb their prevalence to prevent environmental contamination. Furthermore, the discovery of 18 novel genotypes adds to our understanding of the vast genetic variations within *E. bieneusi*. Additional research is needed to further unlock the mysteries of this parasite’s genetic diversity.

## Figures and Tables

**Table 1:** Prevalence of *E. bieneusi* in the investigated shrews and wild rats by species, season, gender, and location.

Category	Positive/Examined (%)							*p*-Value
	*Suncus murinus*	*Apodemus agrarius*	*Niviventer niviventer*	*Rattus losea*	*Rattus norvegicus*	*Rattus tanezumi*	Total	
Sampling site								*p* < 0.001
Ruian	6/147 (4.1)	8/36 (22.2)	4/51 (7.8)	5/18 (27.8)	9/100 (9.0)	1/36 (2.8)	33/388 (8.5)	
Yongjia	26/71 (36.6)	/	2/24 (8.3)	/	2/41 (4.9)	5/34 (14.7)	35/170 (20.6)	
Yueqing	19/64 (29.7)	/	/	/	2/14 (14.3)	3/16 (18.8)	24/94 (25.5)	
Season								*p* < 0.001
Spring	12/82 (14.6)	2/17 (11.8)	1/22 (4.5)	3/7 (42.9)	3/38 (7.9)	1/16 (6.3)	22/182 (12.1)	
Summer	39/96 (40.6)	1/7 (14.3)	5/26 (19.2)	0/3	10/88 (11.4)	7/54 (13.0)	62/274 (22.6)	
Autumn	0/104	5/12 (41.7)	0/27	2/8 (25.0)	0/29	1/16 (6.3)	8/196 (4.1)	
Gender								
Female	43/119 (36.1)	3/15 (20.0)	4/46 (8.7)	3/4 (75.0)	9/86 (10.5)	6/25 (24.0)	68/295 (23.1)	*p* < 0.001
Male	8/163 (4.9)	5/21 (23.8)	2/29 (6.9)	2/14 (14.3)	4/69 (5.8)	3/61 (4.9)	24/357 (6.7)	
Total	51/282 (18.1)	8/36 (22.2)	6/75 (8.0)	5/18 (27.8)	13/155 (8.4)	9/86 (10.5)	92/652 (14.1)	* *p* = 0.007

* *p* = a comparative analysis among six distinct animal species.

**Table 2:** Distribution of *E. bieneusi* genotypes in shrews and wild rats, stratified by species, season, gender, and location.

Category	*E. bieneusi* Genotype (n)
Animal species	
*Suncus murinus*	HNR-VI (28), Peru 11 (6), TypeIV (2), KIN-1 (2), HNPL-II (2), Macaque4 (1), HNHZ-III (1), HNPL-I (1), HNPL-III (1), CH5 (1), Henan-V (1), WZSH-I to V (one each)
*Apodemus agrarius*	WZR-VIII (3), HNP-II (1), WZR-IX to WZR-XII (one each)
*Niviventer niviventer*	HNR-IV (4), WZR-VIII (2)
*Rattus losea*	HNR-IV (5)
*Rattus norvegicus*	HNR-VI (2), SHW7 (2), HNHZ-II (1), K (1), WZR-I to VII (one each)
*Rattus tanezumi*	HNR-VI (8), Henan-III (1)
Season	
Spring	HNR-VI (10), Peru 11 (4), HNPL-I (1), HNPL-II (1), HNPL-III (1), HNHZ-II (1), WZR-I (1), WZR-IV (1), WZSH-V (1), Henan-V (1)
Summer	HNR-VI (33), WZR-VIII (5), Peru 11 (2), TypeIV (2), KIN-1 (2), SHW7 (2), Macaque4 (1), CH5 (1), K (1), Henan-III (1), Henan-III (1), Henan-V (1), HNP-II (1), HNPL-II (1), HNHZ-III (1), WZR-II (1), WZR-III (1), WZR-V (1), WZR-VI (1), WZR-VII (1), WZR-IX (1), WZR-X (1), WZR-XI (1), WZR-XII (1)
Autumn	HNR-VI (4), WZSH-I to IV (one each)
Gender	
Female	HNR-VI (34), WZR-VIII (5), Peru 11 (4), TypeIV (2), Macaque4 (1), CH5 (1), K (1), Henan-III (1), HNP-II (1), HNPL-II (1), HNHZ-III (1), WZR-III (1), WZR-V (1), WZR-VI (1), WZR-VII (1), WZR-IX (1), WZR-X (1), WZR-XI (1), WZR-XII (1), WZR-I (1), WZR-IV (1), WZSH-V (1), Henan-V (1), WZSH-I to IV (one each)
Male	HNR-VI (13), Peru 11 (2), KIN-1 (2), SHW7 (2), HNPL-II (1), HNPL-I (1), HNPL-III (1), HNHZ-II (1), WZR-II (1)
Location	
Yueqing	HNR-VI (14), Peru 11 (4), WZR-VIII (3), WZSH-V (1), HNPL-III (1), HNHZ-II (1)
Yongjia	HNR-VI (14), KIN-1 (2), Peru 11 (2), SHW7 (2), TypeIV (2), Macaque4 (1), CH5 (1), Henan-V (1), HNPL-II (1), HNPL-I (1), WZR-I to WZR-VIII (one each)
Rui’an	HNR-VI (19), K (1), Henan-III (1), HNP-II (1), HNPL-II (1), HNHZ-III (1), WZR-VIII to WZR-XII (one each), WZSH-I to IV (one each),
Total	HNR-VI (47), Peru 11 (6), WZR-VIII (5), TypeIV (2), KIN-1 (2), SHW7 (2), HNPL-II (2), Macaque4 (1), CH5 (1), K (1), Henan-III (1), Henan-V (1), HNP-II (1), HNPL-I (1), HNPL-III (1), HNHZ-II (1), HNHZ-III (1), WZR-I to VII (one each), WZR-IX to WZR-XII (one each), WZSH-I to V (one each)

**Table 3:** Similarity analysis of the novel genotypes of *E. bieneusi* identified in the present study.

Genotype	Accession No.	Accession No-Genotype	Similarity (%)	Position (Nucleotide)
WZR-I	PP391796	MN056209-JLD-XVII	97.5	30 (T to C), 33 (A to G), 58 (T to G), 184 (G to A), 189 (G to T), 191 (T insert)
WZR-II	PP391797	MK322761-CHG13	98.8	116 (T to G), 128 (A to G), 224 (G to T)
WZR-III	PP391798	MT4584689-SHW7	99.2	46 (T to A), 109 (A to G)
WZR-IV	PP391799	MT4584689-SHW7	99.6	119 (T to C)
WZR-V	PP391800	MT4584689-SHW7	99.2	111 (T to C), 138 (C to T)
WZR-VI	PP391801	MH714712-K	96.3	56 (G to A), 75 (G to A), 76 (T insert), 79 (G to C), 96 (G to A), 97 (T insert), 104 (G to C), 109 (T to A), 115 (T to A)
WZR-VII	PP391802	MH714712-K	97.9	75 (A to C), 116 (A to T), 125 (G to T), 181 (A to C), 239 (A to C)
WZR-VIII	PP391803	MN267057-HNR-VI	99.6	14 (G insert)
WZR-IX	PP391804	MN267057-HNR-VI	99.2	14 (G insert), 138 (T to C)
WZR-X	PP391805	MN267057-HNR-VI	98.8	14 (G insert), 132 (C to G), 138 (T to C)
WZR-XI	PP391806	KJ475402-HLJ-I	99.2	10 (G to A), 158 (T to G)
WZR-XII	PP391807	KP732476-NESH2	81.4	45 base difference
WZSH-I	PP391808	MT796858-D	98.8	130 (G to A), 164 (G to T), 175 (A to G)
WZSH-II	PP391809	MT804374-EbpC	99.6	10 (A insert)
WZSH-III	PP391810	KR8155141-KIN-1	98.4	48 (T to C), 81 (T to C),141 (C to T), 179 (C to T)
WZSH-IV	PP391811	MH817463-CHG19	99.6	141 (C to T)
WZSH-V	PP391812	MN845067-Peru11	99.6	202 (T to C)

## Data Availability

Data are contained within the article.
